# Hepatitis C virus NS5A protein blocks epidermal growth factor receptor degradation via a proline motif- dependent interaction

**DOI:** 10.1099/vir.0.000145

**Published:** 2015-08

**Authors:** Zsofia Igloi, Arunas Kazlauskas, Kalle Saksela, Andrew Macdonald, Jamel Mankouri, Mark Harris

**Affiliations:** ^1^​ School of Molecular and Cellular Biology, Faculty of Biological Sciences, University of Leeds, Leeds LS2 9JT, UK; ^2^​ Department of Virology, Haartman Institute, University of Helsinki and Helsinki University Central Hospital, Helsinki 00014, Finland

## Abstract

Hepatitis C virus (HCV) establishes a persistent infection that in many cases leads to cirrhosis and hepatocellular carcinoma. The non-structural 5A protein (NS5A) has been implicated in this process as it contains a C-terminal polyproline motif (termed P2) that binds to Src homology 3 (SH3) domains to regulate cellular signalling and trafficking pathways. We have shown previously that NS5A impaired epidermal growth factor (EGF) receptor (EGFR) endocytosis, thereby inhibiting EGF-stimulated EGFR degradation by a mechanism that remained unclear. As EGFR has been implicated in HCV cell entry and trafficking of the receptor involves several SH3-domain containing proteins, we investigated in more detail the mechanisms by which NS5A perturbs EGFR trafficking. We demonstrated that the P2 motif was required for the NS5A-mediated disruption to EGFR trafficking. We further demonstrated that the P2 motif was required for an interaction between NS5A and CMS, a homologue of CIN85 that has previously been implicated in EGFR endocytosis. We provided evidence that CMS was involved in the NS5A-mediated perturbation of EGFR trafficking. We also showed that NS5A effected a loss of EGFR ubiquitination in a P2-motif-dependent fashion. These data provide clues to the mechanism by which NS5A regulates the trafficking of a key cellular receptor and demonstrate for the first time the ability of NS5A to regulate host cell ubiquitination pathways.

## Introduction

Hepatitis C virus (HCV) is an enveloped virus with a positive-sense, ssRNA genome belonging to the genus *Hepacivirus* within the family *Flaviviridae*. The 9.6 kb HCV genome encodes a single large polyprotein of 3000 aa which is processed co- and post-translationally by viral and host cellular proteases into 10 functional proteins. These include three structural proteins (core, E1 and E2) and seven non-structural proteins (p7, NS2, NS3, NS4A, NS4B, NS5A and NS5B) (Scheel & Rice, 2013).

**Fig. 1.**
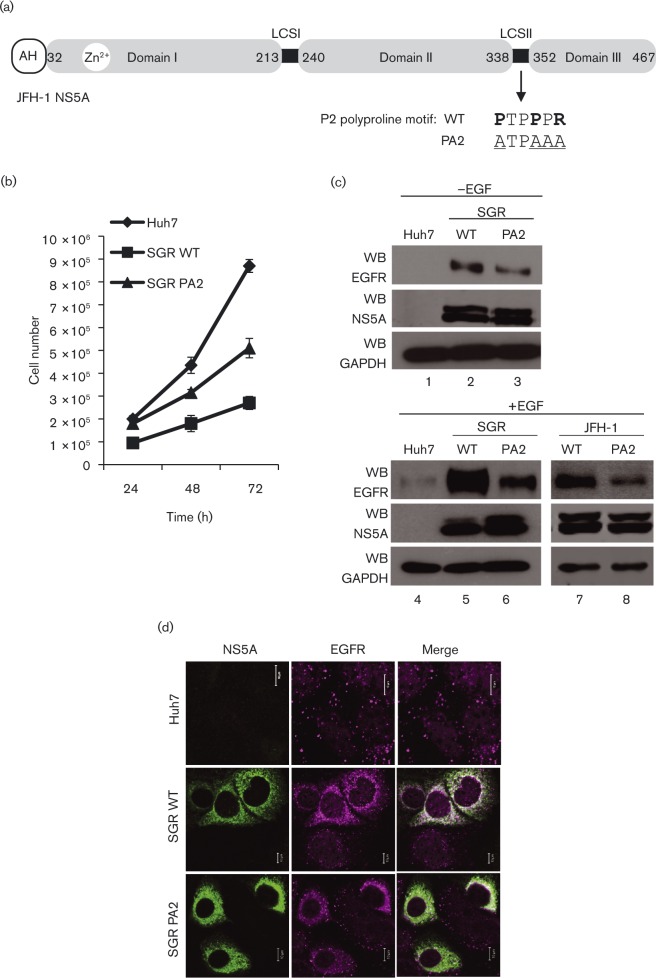
NS5A increases EGFR levels in a polyproline motif-dependent manner. (a) Schematic representation of the domain structure of NS5A. Numbers represent amino acid positions from JFH-1 (genotype 2a). The P2 polyproline motif in LCSII between residues 338 and 352 is indicated. Residues in bold show the conserved prolines and arginine in P2, and underlining shows those residues substituted by alanine in the PA2 mutant. AH, amphipathic helix. (b) Samples of 1 × 10^5^ control Huh7 cells, or cells harbouring either WT or PA2 SGR-neo-JFH-1, were seeded in six-well plates. Cells were trypsinized and counted at 24, 48 and 72 h post-seeding. (c) Control Huh7 cells (lanes 1, 4), cells harbouring either WT (lanes 2, 5) or PA2 (lanes 3, 6) SGR-neo-JFH-1, or cells electroporated with 10 μg JFH-1 virus RNA (WT or PA2 mutant, lanes 7, 8) were serum starved overnight and either stimulated with 50 ng EGF ml^− 1^ for 30 min or left unstimulated. Lysates were Western blotted (WB) for endogenous EGFR, NS5A and glyceraldehyde 3-phosphate dehydrogenase (GAPDH). (d) Control Huh7 cells, or cells harbouring either WT or PA2 SGR-neo-JFH-1, were serum starved overnight and stimulated with 50 ng EGF ml^− 1^ for 30 min prior to fixation and permeabilization. Cells were stained for NS5A (green) and endogenous EGFR (magenta), and analysed by confocal microscopy. Bar, 10 μm.

One of these non-structural proteins, NS5A, is an RNA-binding phosphoprotein essential for both viral replication and viral particle assembly (Ross-Thriepland & Harris, 2015). It is composed of an N-terminal amphipathic helix that mediates membrane association (Elazar *et al.*, 2003) followed by three domains separated by two low-complexity sequences (LCSs) (Tellinghuisen *et al.*, 2004). The structure of domain I has been determined (Lambert *et al.*, 2014; Love *et al.*, 2009; Tellinghuisen *et al.*, 2005) and this domain binds to RNA (Foster *et al.*, 2010; Huang *et al.*, 2005). Domain II is required for RNA replication (Ross-Thriepland *et al.*, 2013) and domain III is involved in virion assembly (Appel *et al.*, 2008; Hughes *et al.*, 2009a). The LCS between domains II and III is proline-rich (Fig. 1a) and, of note, contains a highly conserved PxxPxR sequence (Macdonald *et al.*, 2004, 2005b; Tan *et al.*, 1999). This sequence matches the consensus for a class II SH3-domain-binding motif (Mayer, 2001) and is referred to herein as the P2 motif. Several cellular SH3-domain-containing binding partners for the P2 motif have been identified (Macdonald *et al.*, 2004; Nanda *et al.*, 2006), but their roles in the HCV life cycle remain undefined.

NS5A is known to coordinate key interactions among viral and host proteins to aid virus pathogenesis (Ross-Thriepland & Harris, 2015). Our previous work demonstrated that NS5A could disrupt the trafficking and degradation of the epidermal growth factor receptor (EGFR) during HCV infection (Macdonald *et al.*, 2005a; Mankouri *et al.*, 2008). We demonstrated that this effect was not due to direct binding of NS5A to the EGFR, but the mechanism remained unclear. The EGFR has emerged as a key HCV entry factor (Diao *et al.*, 2012; Lupberger *et al.*, 2011) and EGFR signalling has been shown to influence HCV replication (Lupberger *et al.*, 2013). Therefore, to fully define the role of NS5A in these processes, we set out to investigate the mechanism by which HCV NS5A disrupts EGFR trafficking and the cellular co-factors by which NS5A mediates this effect.

We show here that the P2 motif of NS5A is required for the disruption of EGFR trafficking, implicating interactions between NS5A and cellular SH3-domain-containing proteins in this process. We identify the ubiquitously expressed adaptor molecule, Cas ligand with multiple SH3 domains (CMS) [also known as CD2-associated protein (CD2AP)], as an SH3-domain-containing NS5A-interacting protein and a candidate for a role in NS5A-mediated perturbation of EGFR trafficking. We further demonstrate that NS5A disrupts EGFR ubiquitination in a P2-dependent manner. These data provide a mechanistic insight detailing how NS5A controls EGFR trafficking and provides the first evidence that NS5A can regulate host cell ubiquitination.

## Results

### NS5A inhibits EGFR degradation in a P2-motif-dependent fashion

EGFR is known to undergo clathrin-mediated endocytosis preceding early to late endosomal trafficking and subsequent degradation (Zheng *et al.*, 2014). We have previously shown that NS5A partially localized to early endosomes and, although it had no effect on EGF internalization, colocalized with the EGFR and altered its distribution (Mankouri *et al.*, 2008). This redistribution correlated with a decrease in the amount of active EGF–EGFR ligand–receptor complexes present in the late endosomal signalling compartment and also resulted in a concomitant increase in the total levels of EGFR. The mechanism(s) by which NS5A mediated this effect were not elucidated; however, we reasoned that the P2 motif might play a role in this effect by mediating interactions with host cell factor(s). As we have previously shown that this motif was not required for virus replication (Hughes *et al.*, 2009b), we were therefore able to evaluate the cellular functions of the P2 motif in Huh7 cells either stably harbouring subgenomic replicons (SGRs) or infected with JFH-1 virus in which the motif had been disrupted by alanine substitution (PA2).

When the ligand-bound EGFR reaches the late endosomal compartment it activates a number of signalling cascades leading to stimulation of cell growth and division. One consequence of NS5A perturbation of EGFR trafficking might therefore be a reduction in the rate of cell growth. This is indeed what we observed (Fig. 1b); whereas the doubling time of naïve Huh7 cells was ∼24 h, those harbouring the WT SGR exhibited a doubling time of ∼48 h. Consistent with a role for the P2 motif in perturbing EGFR signalling, the growth rate of cells harbouring a PA2 mutant SGR was partially restored and they exhibited an intermediate phenotype.

To assess this phenotype biochemically we therefore examined the consequences of the PA2 mutation on EGFR turnover. A comparison of control Huh7 cells and those expressing NS5A, both in the context of a SGR or infected with HCV, revealed a decrease in the rate of EGFR degradation and enhanced EGFR levels both prior to and post-EGF stimulation (Fig. 1c, compare lanes 1/2 and 4/5). Interestingly the PA2 mutant exhibited a reduction in EGFR levels compared with WT, suggesting that this mutant impaired the ability of NS5A to block EGFR degradation (Fig. 1c, compare lanes 2/3, 5/6 and 7/8). Of note, the PA2 mutant exhibited an intermediate phenotype as levels of EGFR were not as low as in naïve Huh7 cells (Fig. 1c, compare lanes 1–3 and 4–6), consistent with the intermediate growth phenotype in Fig. 1(b). The same phenomenon was also observed with immunofluorescence, staining for endogenous EGFR and NS5A (Fig. 1d). The intermediate phenotype seen with the PA2 mutant suggested that whilst the P2 motif was necessary for perturbation of EGFR endocytosis by NS5A, it was not sufficient and other interactions contributed to the process.

**Fig. 2.**
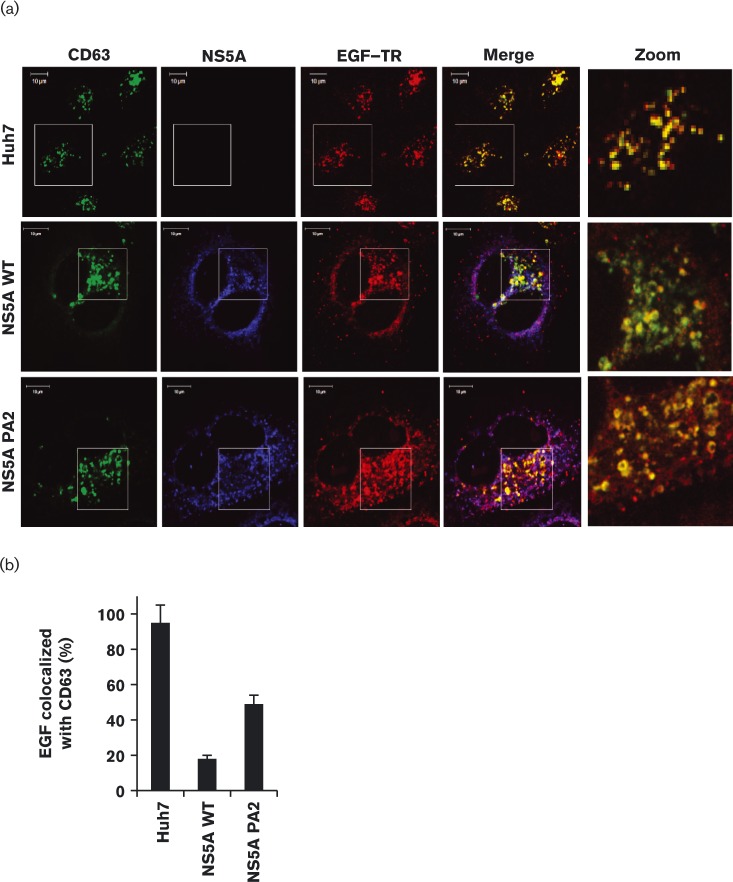
PA2 mutation partially restores EGFR translocation to late endosomal compartments. (a) Mock-transfected Huh7 cells, or cells transfected with plasmids expressing either WT or PA2 NS5A (Macdonald *et al.*, 2004), were serum-starved overnight prior to stimulation with 100 ng EGF–TR ml^− 1^ for 20 min. Cells were then fixed, permeabilized and probed for CD63 using a mouse anti-CD63 antibody followed by an Alexa Fluor 488-conjugated anti-mouse secondary (green) and NS5A using anti-sheep NS5A followed by Alexa Fluor 633 (blue). Cells were fixed and analysed by confocal microscopy. Bar, 10 μm. (b) Percentage of EGF–TR translocation to late endosomes was quantified by staining for CD63 and analysis using Bitplane software as described previously (Mankouri *et al.*, 2008).

Following EGF stimulation, a high proportion of internalized EGFR colocalized with the late endosomal marker CD63. We previously showed that, in NS5A-expressing cells, there was a significant decrease in the intracellular accumulation of EGFR in CD63^+^ late endosomes (Mankouri *et al.*, 2008). Using Texas red (TR)-conjugated EGF to track the endocytosis of EGFR we confirmed that, in control cells, ∼85 % of EGF colocalized with CD63, whereas this was reduced to 15 % in cells expressing NS5A (Fig. 2a, b). In cells expressing PA2 mutant NS5A, EGF translocation to the late endosomal compartment was partially restored; 46 % of the EGF colocalized with CD63, again consistent with the intermediate phenotype of this mutant shown in Fig. 1. This further demonstrated a critical role for the NS5A P2 motif in the disruption of EGFR trafficking and subsequent degradation, but again implicated a role for other interactions mediated by NS5A in a non-P2-motif-dependent fashion.

### NS5A binds to CMS, a known mediator of EGFR endocytosis

**Fig. 3.**
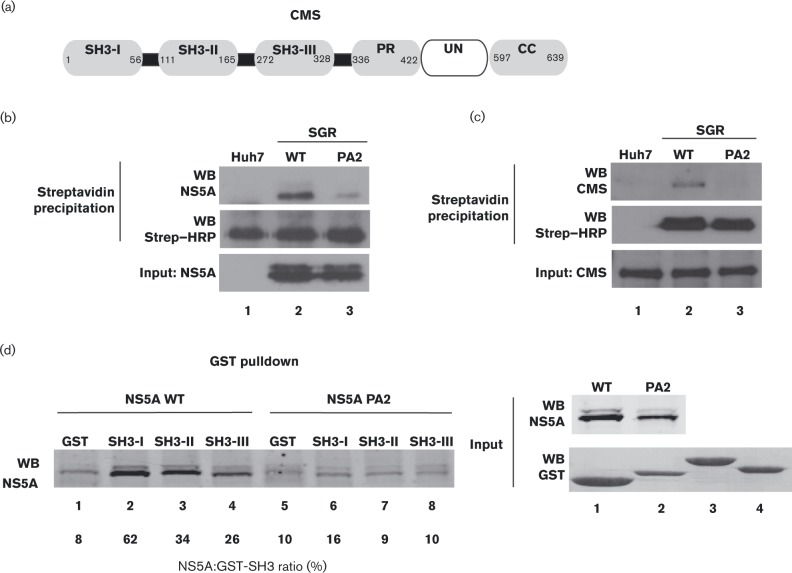
CMS specifically binds to the P2 motif in NS5A. (a) Schematic of the domain structure of CMS. CMS is composed of three N-terminal SH3 domains (SH3-I, -II and -III), followed by a proline-rich (PR) region, an unstructured region of ∼160 residues (UN) and a C-terminal coiled-coil (CC) domain. Numbers represent amino acid positions. (b) Control Huh7 cells, or WT or PA2 SGR-harbouring cells, were transfected with a plasmid expressing CMS–PP. At 36 h post-transfection the cells were serum-starved overnight prior to stimulation with 50 ng EGF ml^− 1^ for 30 min. Lysates were precipitated using streptavidin beads and the eluted fractions analysed by Western blotting (WB) with an antibody to NS5A or streptavidin–HRP (Strep–HRP; to detect CMS–PP). (c) Control Huh7 cells, or cells harbouring WT or PA2 SGR-neo-JFH-1 in which NS5A was tagged with the ΔPSTCD-tag (McCormick *et al.*, 2006), were serum-starved overnight prior to stimulation with 50 ng EGF ml^− 1^ for 30 min. Lysates were precipitated using streptavidin beads. The eluted fractions were analysed by Western blotting (WB) with antibody to endogenous CMS or streptavidin–HRP (Strep–HRP; to detect NS5A-ΔPSTCD). (d) Purified GST or GST-tagged CMS SH3 domains (SH3 I, II and III for the three SH3 domains in CMS, respectively) were bound to glutathione-agarose beads and incubated overnight with lysates from cells harbouring WT or PA2 SGR-neo-JFH-1.The eluted fractions were separated on SDS-PAGE and Western blotted (WB) for NS5A; 15 % of the amount used for the pulldown experiment was loaded as input. Densitometry analysis of bound NS5A is displayed as a percentage of bound NS5A: GST-SH3 ratio underneath the Western blot.

EGFR trafficking is dependent on its coordinated interaction with several SH3-domain-containing proteins (Zheng *et al.*, 2014). Many SH3 domains have been shown to bind to NS5A in a P2-motif-dependent fashion (Macdonald *et al.*, 2004; Nanda *et al.*, 2006; Tan *et al.*, 1999) and we therefore exploited a phage display library expressing all 296 SH3 domains in the human genome (Kärkkäinen *et al.*, 2006) to identify NS5A interactors that might explain the NS5A-mediated effects on EGFR trafficking. We generated WT and PA2 mutant JFH-1-derived SGRs in which a truncated form of the *Propionibacterium shermanii* transcarboxylase domain (ΔPSTCD) was inserted into domain III of NS5A to express an NS5A-ΔPSTCD fusion protein. This tag acted as a biotin acceptor allowing streptavidin-based purification of the NS5A-ΔPSTCD fusion protein (McCormick *et al.*, 2006). WT and PA2 NS5A-ΔPSTCD were purified from SGR-harbouring cell lysates using streptavidin-magnetic beads and used to screen the SH3 domain phage display library as described (Neuvonen *et al.*, 2011). The SH3 domains of a number of previously reported NS5A-binding partners, including the Src kinases Hck, Lyn, Src (Macdonald *et al.*, 2004; Shelton & Harris, 2008) and Amphiphysin II (Amph2, also known as Bin1) (Nanda *et al.*, 2006), were identified. In addition, several novel interacting partners were discovered, including mixed-lineage kinase (Mlk3), further characterization of which is described elsewhere (Amako *et al.*, 2013), and the CIN85 homologue, CMS (Dikic, 2002) (Fig. 3a). This was of interest as both CIN85 and CMS have been implicated in EGFR endocytosis (Kirsch *et al.*, 2001). To validate the result of the phage screen, the interaction of NS5A with CMS was confirmed by co-immunoprecipitation. WT or PA2 SGR-harbouring cells were transfected with a plasmid expressing CMS with a C-terminal PinPoint (PP) tag (a commercially available variant of PSTCD), and lysates were precipitated with streptavidin-magnetic beads and analysed by Western blotting. This analysis showed that CMS bound to WT NS5A, but this interaction was reduced by introduction of the PA2 mutation (Fig. 3b). Furthermore, NS5A expressed from the ΔPSTCD-containing SGR also interacted with endogenous CMS in a P2-motif-dependent fashion (Fig. 3c).

CMS contains three SH3 domains (Fig. 3a); the phage display screen identified that domains I and II mediated its interaction with WT NS5A. To confirm this, the individual SH3 domains of CMS were expressed as glutathione *S*-transferase (GST) fusions, purified and used in GST-pulldown experiments with lysates from either WT or PA2 SGR-harbouring cells. Fig. 3(d) shows that, although WT but not PA2 NS5A bound to all three SH3 domains, a clear preference for domains I and II existed. Taken together, these data verified CMS as a novel interacting partner of HCV NS5A.

### CMS can rescue EGFR degradation during HCV infection

**Fig. 4.**
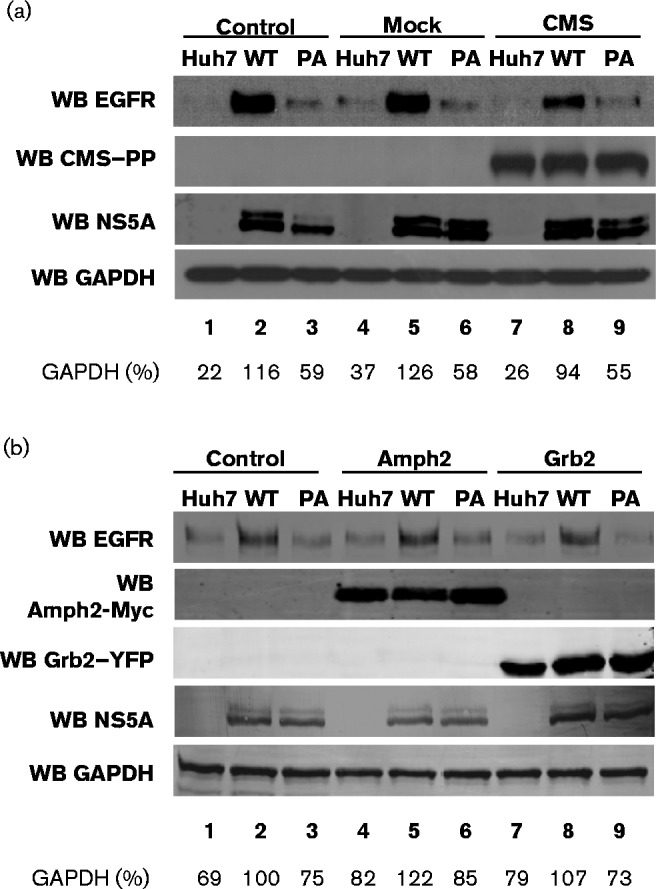
CMS can partially rescue EGFR degradation in WT NS5A-containing cells. Control Huh7 cells, or cell harbouring WT or PA2 SGR-neo-JFH-1, were transfected with plasmids expressing (a) CMS–PP or (b) Amph2–HA or Grb2–YFP and at 36 h post-transfection the cells were serum starved overnight prior to stimulation with 50 ng EGF ml^− 1^ for 30 min. Lysates were Western blotted (WB) for endogenous EGFR, CMS–PP (using the streptavidin–HRP antibody), NS5A, GAPDH, Amph2-Myc (using the Myc epitope antibody) or Grb2–YFP (using the GFP antibody) as indicated. Densitometry analysis of EGFR levels is displayed as a normalized percentage of GAPDH loading control underneath the Western blots.

We next sought to assess the involvement of the NS5A–CMS interaction in EGFR trafficking. CMS was expressed as a fusion with the PP tag (CMS–PP) in WT and PA2 SGR-harbouring cells, and EGFR levels were analysed by Western blotting. In control samples (non-transfected and empty vector), a blockade of EGFR degradation by NS5A post-EGF stimulation (Fig. 4a, lanes 2 and 5) was observed. The PA2 SGR exhibited an intermediate phenotype (Fig. 4a, lanes 3 and 6), consistent with the data shown in Fig. 1(c). Interestingly, following exogenous expression of CMS–PP, EGF-mediated EGFR degradation was restored in WT NS5A-expressing cells, where a clear loss of EGFR levels was observed (Fig. 4a, lane 8). This was not the case when two other SH3 domain-containing proteins, Amph2-Myc and Grb2–YFP (yellow fluorescent protein), also known to participate in EGFR endocytosis and trafficking, and to interact with NS5A, were exogenously expressed (Fig. 4b, lanes 5 and 8). These data demonstrated that CMS could specifically restore EGFR degradation in NS5A-expressing cells, implicating the NS5A–CMS interaction as a key event governing this process. Intriguingly, ablation of CMS expression by small interfering RNA had no effect on EGFR degradation in either control Huh7 cells or SGR-harbouring cells (data not shown). This could be explained by the fact that CMS shares overlapping binding specificities and biological functions with CIN85 – an adaptor that also plays an essential role in EGFR lysosome trafficking and degradation. Thus, CIN85 might be able to functionally compensate for the lack of CMS.

### NS5A mediates a loss of EGFR ubiquitination following EGF stimulation

CIN85, together with the ubiquitin ligase casitas B-lineage lymphoma (c-Cbl), is known to be vital for the ubiquitination of EGFR (Eden *et al.*, 2012; Haglund *et al.*, 2002; Kirsch *et al.*, 2001; Soubeyran *et al.*, 2002; Szymkiewicz *et al.*, 2002). Although partial ubiquitination of the EGFR occurs at the cell surface (Stang *et al.*, 2000), it is not required for internalization (Huang *et al.*, 2007). EGFR ubiquitination is instead postulated to be required for the progression from early endosomes to late endosomes/multivesicular bodies (Grøvdal *et al.*, 2004; Huang *et al.*, 2013). Given this body of evidence, we reasoned that EGFR ubiquitination might be the pathway altered by NS5A.

**Fig. 5.**
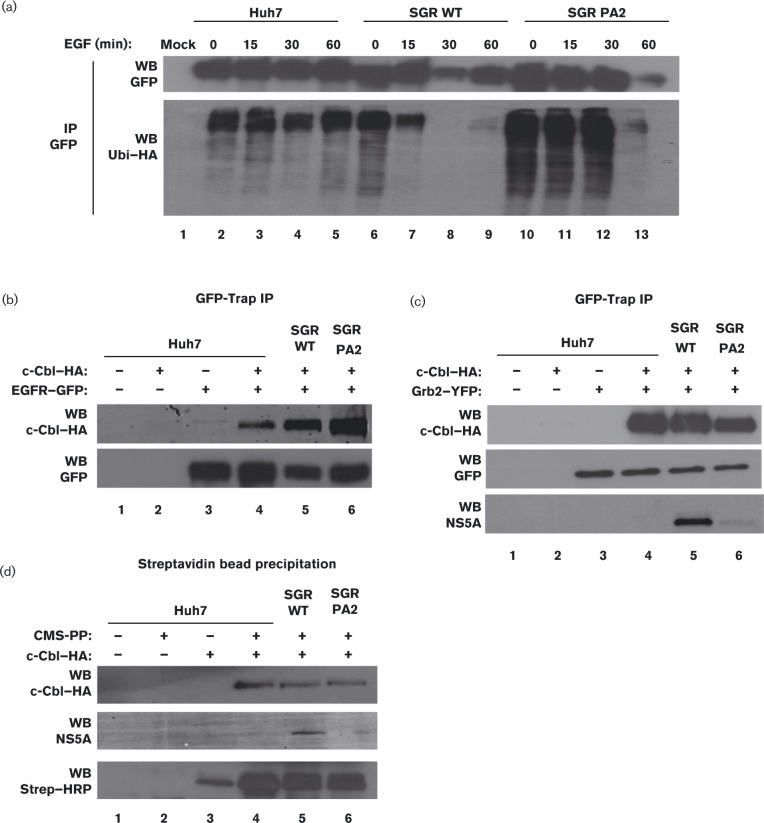
NS5A mediates a loss of EGFR ubiquitination and degradation. (a) Control Huh7 cells, or cells harbouring WT or PA2 SGR-neo-JFH-1, were transiently transfected with plasmids expressing EGFR–GFP and ubiquitin–HA (Ubi–HA). At 36 h post-transfection the cells were serum starved overnight prior to stimulation with 50 ng EGF ml^− 1^ for 0, 15, 30 or 60 min. Lysates were immunoprecipitated (IP) using GFP-Trap beads, and eluted fractions were separated on SDS-PAGE and probed with antibodies to HA (to detect Ubi–HA) or GFP (to detect EGFR–GFP). Mock, untransfected Huh7 cells (Lane 1). (b) Control Huh7 cells, or cells harbouring WT or PA2 SGR-neo-JFH-1, were transfected with plasmids expressing EGFR–GFP and Cbl–HA as indicated. At 36 h post-transfection the cells were processed as in (a), prior to Western blotting (WB) for HA or GFP. (c) Control Huh7 cells, or cells harbouring WT or PA2 SGR-neo-JFH-1, were transiently transfected with plasmids expressing Grb2–YFP and c-Cbl–HA as indicated. At 36 h post-transfection the cells were processed as in (a), prior to Western blotting (WB) for HA, YFP (using the GFP antibody) or NS5A. (d) Control Huh7 cells, or cells harbouring WT or PA2 SGR-neo-JFH-1, were transfected with plasmids expressing CMS–PP and c-Cbl–HA as indicated. At 36 h post-transfection the cells were processed as in (a), prior to precipitation of lysates using streptavidin beads. The eluted fractions were separated on SDS-PAGE and probed with antibodies to HA or NS5A, or with streptavidin–HRP (Strep–HRP; to detect CMS–PP).

To test this, control or SGR-harbouring cells were transfected with plasmids expressing haemagglutinin (HA)-tagged ubiquitin (Ubi–HA) and an EGFR–GFP fusion. After serum starvation and EGF stimulation, EGFR–GFP was immunoprecipitated using the GFP-Trap system and immunoprecipitates were Western blotted for the presence of Ubi–HA. When compared with control Huh7 cells, WT SGR-harbouring cells exhibited a significant loss of receptor ubiquitination from 0 to 60 min post-EGF stimulation (Fig. 5a, compare lanes 2–5 and 6–9). Furthermore, the PA2 mutation restored receptor ubiquitination post-EGF stimulation to levels similar to those of control Huh7 cells (Fig. 5a, lanes 10–13). No signal was observed in mock-transfected Huh7 cells (Fig. 5a, lane 1). These data suggested that in response to ligand stimulation, NS5A mediated a rapid loss of EGFR ubiquitination. Intriguingly, unlike the intermediate phenotype of the PA2 mutant observed previously (Figs. 1 and 2), in this case the mutation resulted in a complete restoration of EGFR ubiquitination. This was consistent with the proposal that NS5A interacted with the EGFR endocytic pathway at multiple stages and that these may involve different interactions of the viral protein with cellular factors, not all of which are mediated via P2 binding to SH3 domains.

It has previously been reported that EGFR ubiquitination and subsequent degradation is disrupted by perturbing complex formation between CIN85 and c-Cbl (Soubeyran *et al.*, 2002; Szymkiewicz *et al.*, 2002). c-Cbl can bind to the EGFR either directly or indirectly via Grb2 in an SH3 domain-directed fashion (Grøvdal *et al.*, 2004). We further investigated the interaction of c-Cbl with EGFR, Grb2 and CMS in either control Huh7 cells or those harbouring either WT or PA2 SGR to define the molecular events underpinning the effects of NS5A. First, the ability of c-Cbl to interact with EGFR either directly or via Grb2 was tested. Cells were transiently transfected with plasmids expressing EGFR–GFP and HA-tagged c-Cbl (Cbl–HA), and the lysates were immunoprecipitated using the GFP-Trap system and probed for the presence of Cbl–HA (Fig. 5b). Western blot analysis revealed that c-Cbl was able to interact with EGFR and this interaction was unaffected by the presence of the SGR (WT or PA2) (Fig. 5b, lanes 4–6). In a similar experiment, cells were transfected with plasmids expressing YFP-tagged Grb2 and Cbl–HA. Western blot analysis of the GFP-Trap precipitated fractions showed that c-Cbl was able to interact with Grb2 and this interaction was again unaffected by the SGR (Fig. 5c, lanes 4–6). As described previously (Macdonald *et al.*, 2005a), WT NS5A, but not the PA2 mutant, precipitated with Grb2 (Fig. 5c, lanes 5 and 6). The presence of both NS5A and c-Cbl in the immunoprecipitated fractions suggested that NS5A and c-Cbl did not compete for a single binding site on Grb2 and may co-interact with each of the two Grb2 SH3 domains.

As NS5A did not appear to disrupt the interaction between c-Cbl, Grb2 and EGFR, we next assessed whether the effect on EGFR ubiquitination could be explained by the effects of NS5A on the interactions between the adaptor CMS and either EGFR or c-Cbl. Control or SGR-harbouring Huh7 cells were transfected with plasmids expressing c-Cbl–HA and CMS–PP. CMS–PP was precipitated using streptavidin-magnetic beads and Western blotted for the presence of Cbl–HA and NS5A. As shown in Fig. 5(d), c-Cbl was able to bind to CMS in both the absence and presence of NS5A (Fig. 5d, lanes 4–6), suggesting that the c-Cbl–CMS interaction remained unaffected. Consistent with the GST-pulldown data, CMS–PP did not interact with the PA2 mutant NS5A (Fig. 5d, lane 6). Taken together, these data indicated that the SH3-binding domain of NS5A modulated the ubiquitination status of EGFR, but this was not due to the inhibition of c-Cbl–EGFR binding.

## Discussion

**Fig. 6.**
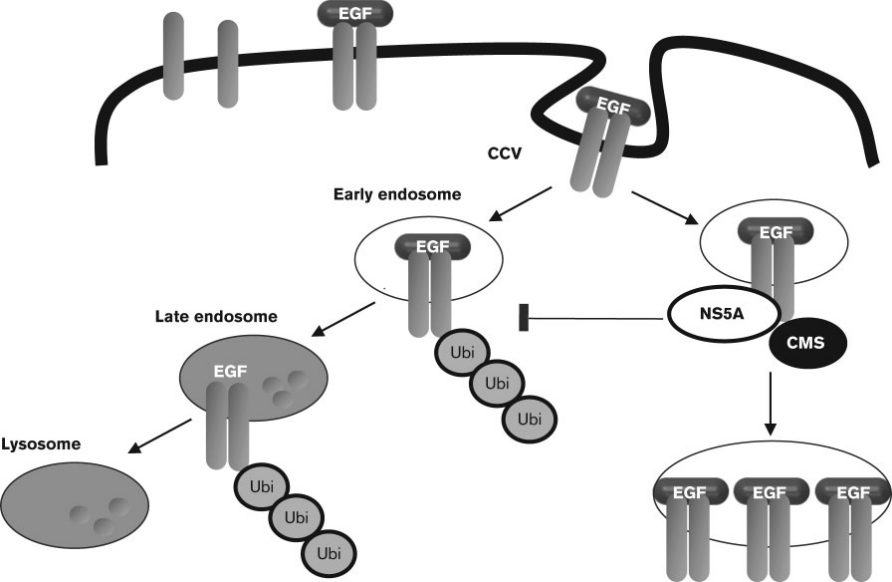
Proposed schematic of EGFR trafficking in the presence of NS5A. Upon EGF stimulation, EGFR enters clathrin-coated vesicles (CCV). Following internalization, EGFR initially traffics through early endosomes, where NS5A was also shown to partially localize (Mankouri *et al.*, 2008). Activation of the receptor results in EGFR ubiquitination (Ubi) and targeting for degradation through late endosomal compartments to lysosomes, as a means to terminate EGFR signalling. In the presence of NS5A, EGFR ubiquitination is blocked and its subsequent trafficking to late endosomal or lysosomal compartments is delayed by a CMS-dependent mechanism.

It is known that HCV perturbs the cellular environment to favour its own replication; however, much remains unknown about how HCV proteins elicit such cellular changes. In this regard, NS5A has previously been shown to colocalize with the EGFR and alter its distribution during HCV infection (Mankouri *et al.*, 2008). This redistribution correlates with a decrease in the levels of EGF–EGFR ligand–receptor complexes in the late endosomal/lysosomal compartment and results in a concomitant increase in the total levels of EGFR in HCV-infected cells. Here, we investigated the mechanism and host cell proteins important for this effect. Our data indicate that the P2 motif in NS5A is required for perturbation of EGFR trafficking. Ectopic expression of a ligand for the NS5A P2 motif CMS can recover normal EGFR trafficking. Finally, we show that NS5A results in a rapid loss of EGFR ubiquitination following EGF stimulation. A schematic for how we propose NS5A might perturb EGFR trafficking is presented in Fig. 6.

As for other receptor tyrosine kinases (RTKs), the function of EGFR is controlled by intracellular trafficking. The activation of RTKs by growth factors increases turnover and leads to downregulation of the RTK. Although the control of EGFR trafficking per se is neither pro- nor antiviral, EGFR has been assigned several roles in cellular pathways known to be manipulated during HCV infection. These include its role as a cofactor during HCV entry, mediated by CD81–claudin-1 co-receptor association (Diao *et al.*, 2012; Lupberger *et al.*, 2011) and the role of EGFR signalling in the impairment of the antiviral activity of IFN-α in hepatocytes (Lupberger *et al.*, 2013). Controlling EGFR degradation may therefore represent a significant factor in the ability of HCV to avoid host cell immune responses and aid viral spread. In this regard, it is interesting to note that PA2 mutant HCV viruses, which fail to inhibit EGF-stimulated EGFR degradation, did not establish a persistent infection in chimpanzees (Nanda *et al.*, 2006). In cultured Huh7 cells, introduction of the PA2 mutation did not impair virus replication or assembly/release (Hughes *et al.*, 2009b). This may indicate a key role of the NS5A P2 motif in the establishment or maintenance of HCV infection *in vivo.* The contribution of EGFR trafficking and signalling during *in vivo* HCV infection therefore warrants further investigation.

CMS belongs to the family of ubiquitously expressed adaptor molecules that can associate with accessory endocytic proteins as well as other adaptor proteins involved in RTK signalling. Whilst NS5A blocks ligand-induced EGFR degradation through its interaction with this adaptor (Fig. 5a), this did not correlate with changes in the interaction of EGFR with its key downstream signalling proteins (c-Cbl, Grb2 and CMS) (Fig. 5b–d). How the interaction of NS5A with CMS modulates the ubiquitination of EGFR remains to be elucidated. The loss of EGFR ubiquitination seen in Fig. 5(a) is consistent with the active recruitment of a deubiquitinating factor. It is conceivable that this might be accompanied by inhibition of c-Cbl and/or a block to the recruitment of other components of the ubiquitin machinery. Experiments are under way to investigate these hypotheses.

The ability of NS5A to enhance EGFR deubiquitination reaffirms the role of this post-translational modification during EGFR endocytosis. Previous observations reported that when EGFR ubiquitination is inhibited, the receptor is internalized, but not degraded – an effect mirrored during HCV infection (Eden *et al.*, 2012). A further question that remains is the cellular consequence of manipulating EGFR ubiquitination during HCV infection. Numerous lines of epidemiological evidence have indicated that persistent HCV infection is a major risk for the development of hepatocellular carcinoma (HCC). Clinical studies using transgenic mouse models, in which the core or NS5A proteins display oncogenic potential, indicate that HCV proteins are directly involved in HCC development (Koike, 2014). Overexpression of EGFR has been reported in human HCC, and is associated with the late stages of disease, increased cell proliferation and the degree of tumour differentiation (Berasain *et al.*, 2009; Peschard & Park, 2003). EGFR progression through the endocytic pathway is critical for the maintenance of tightly regulated kinase signalling. Indeed, somatically acquired mutations in EGFR, which evade ubiquitin-mediated sorting to degradation, are widespread in cancer. By manipulating EGFR ubiquitination, NS5A may therefore contribute to HCV-mediated HCC development, which adds to the growing evidence linking HCV proteins to HCC formation.

In conclusion, we have provided evidence that NS5A manipulates the ubiquitin system to perturb EGFR trafficking and degradation. The future identification of key molecules in the ubiquitin and proteasome systems that are implicated in these NS5A-mediated processes will provide new insights and an improved understanding of the role of EGFR in HCV pathogenesis – knowledge that is essential for the design of novel anti-HCV therapeutics.

## Methods

### Cell culture

Huh7 cells were cultured in Dulbecco's modified Eagle's medium supplemented with 10 % (v/v) FBS, 100 U penicillin ml^− 1^, 100 μg streptomycin ml^− 1^, 2 mM l-glutamine and non-essential amino acids (Gibco) at 37 °C and 5 % CO_2_ in a humidified incubator. SGR-neo-JFH-1 (Kato *et al.*, 2003) used for this study was derived from the genotype 2a JFH-1 isolate and was obtained from Takaji Wakita (National Institute of Infectious Diseases, Japan). A PA2 mutant of SGR-neo-JFH-1 was generated by QuikChange mutagenesis targeting the LCSII PxxPxR motif of NS5A. SGR-ΔPSTCD-neo-JFH-1 WT and PA2, used in the SH3 domain phage screen, were created by inserting a ΔPSTCD (McCormick *et al.*, 2006) tag into the C terminus of NS5A using an internal *Bcl*I site. Cells stably harbouring SGRs were maintained in the presence of 250 μg G418 ml^− 1^ (Melford). A growth curve was produced by seeding 1 × 10^5^ Huh7, WT and PA2 SGR-containing cells. Cells were trypsinized at 24, 48 and 72 h post-seeding and counted using a haemocytometer. The experiment was carried out twice in duplicate.

### Antibodies and Western blotting

The polyclonal sheep anti-NS5A serum has been described previously (Macdonald *et al.*, 2003). Other antibodies were purchased from the following sources: HA antibody from Sigma; glyceraldehyde 3-phosphate dehydrogenase (GAPDH) and GST from Abcam, CMS #2135 (for Western blots) and #5478 (for immunofluorescence) from Cell Signalling, and GFP and EGFR from Santa Cruz Biotechnology. Myc-tag antibody was generated in house, streptavidin-conjugated HRP was obtained from Thermo Scientific, Texas red-conjugated EGF (EGF–TR) was purchased from Life Technologies, and mouse anti-CD63 was obtained from Abcam. For Western blotting, cells were lysed in Glasgow lysis buffer (GLB) as described previously (Ross-Thriepland & Harris, 2014). Membranes were probed with appropriate primary antibodies followed by either HRP-conjugated secondary antibodies (Sigma) or fluorescent secondary antibodies (LI-COR) and were visualized using an in-house enhanced chemiluminescence reagent or by using a LI-COR Odyssey Sa fluorescent imaging system, respectively. Densitometry analysis was performed on Western blots using ImageJ software.

### Plasmid constructs

Full-length CMS was amplified by PCR with primers containing *Hin*dIII restriction sites. The fragment was directly ligated into a derivative of pcDNA3.1(+) that contained a PinPoint (PP) biotinylation tag inserted via *Bam*HI and *Not*I sites. An Amph2-Myc plasmid was constructed by standard techniques. The NS5A expression plasmid pSG5-NS5A has been described previously (Macdonald*et al.*, 2003). EGFR–GFP was purchased from Addgene. The ubiquitin-coding sequence was amplified with an N-terminal HA-tag using KOD polymerase and cloned into the mammalian expression vector pCMV5 using the *Bam*HI site. Plasmids expressing individual SH3 domains of CMS as GST fusions were obtained from Kathrin Kirsch (Boston University, USA), c-Cbl–HA was obtained from Sreenivasan Ponnambalam (University of Leeds, UK) and Grb2–YFP was obtained from Alexander Sorkin (University of Pittsburgh, USA).

### Transfection of plasmid DNA and immunoprecipitation

Huh7, WT SGR-neo-JFH-1- or PA2 SGR-neo-JFH-1-harbouring cells (1 × 10^6^) were plated in 90 mm dishes and transfected with 5 μg DNA of the expression plasmids (Grb2–YFP and Cbl–HA, EGFR–GFP and Cbl–HA, and EGFR–GFP and ubiquitin–HA; CMS–PP and Cbl–HA) using polyethyleneimine (Sigma-Aldrich). At 36 h post-transfection, cells were serum starved overnight and stimulated with 50 ng EGF ml^− 1^ for 30 min. Cells were lysed in GLB (as described above) and lysates quantified using the bicinchoninic acid protein assay (Thermo Scientific). Lysate (400 μg) was incubated with either GFP-Trap beads (Chromotek) or streptavidin-magnetic beads (Life Technologies) overnight. Beads were washed three times in GLB, eluted by boiling with 10 μl 1 ×  SDS loading buffer and analysed on SDS-PAGE followed by Western blotting. All experiments were repeated a minimum of three times; Western blots displayed are representative.

### Purification of GST–CMS SH3 and GST-pulldown assays

Individual SH3 domains of CMS were expressed as GST fusions and purified. Briefly, pGEX-4T-1-SH3 expression plasmids were grown in *Escherichia coli* BL21(DE3) pLysS at 37 °C until OD_600_ 0.6. The bacteria were induced with 1 mM IPTG at 37 °C for 3 h and then pelleted by centrifugation. Bacteria harvested from 400 ml expression cultures were resuspended in 10 ml PBS, 1 % (v/v) Triton X-100 and protease inhibitors (aprotinin, 2 μg ml^− 1^; leupeptin, 1 μg ml^− 1^; pepstatin A 1 μg ml^− 1^; Pefabloc, 0.4 mM) (Sigma). The bacteria were lysed by sonication and clarified by centrifugation. The clarified lysate was added to freshly prepared GA beads (Sigma) and allowed to bind. The beads were washed twice in PBS plus 1 % (v/v) Triton X-100 at 4 °C and then twice in 50 mM Tris/HCl, pH 8.0. For GST-pulldown experiments, 10 μl GA beads slurry containing either GST or the GST–CMS SH3 fusions was incubated with 400 μg cell lysate of either WT or PA2 SGR-neo-JFH-1 overnight. Beads were washed three times with GLB, eluted by boiling with 10 μl 1 ×  SDS loading buffer and NS5A detected by Western blotting.

### Immunofluorescence

Cells were seeded onto coverslips and serum starved overnight prior to stimulation with 50 ng EGF ml^− 1^ for 30 min. Methanol was used for fixation and methanol/acetone (1: 1) was used for permeabilization. Cells were labelled with sheep polyclonal anti-NS5A antibodies, mouse anti-EGFR, mouse anti-CD63, EGF–TR or rabbit anti-CMS (described above). Alexa Fluor 633- and 488-conjugated donkey anti-sheep, Alexa Fluor 488- and 633-conjugated goat anti-mouse, and Alexa Fluor 594-conjugated goat anti-rabbit fluorescent secondary antibodies (Life Technologies) were used. Overnight serum-starved cells were stimulated with 100 ng EGF–TR ml^− 1^ for 20 min. Cells were then fixed and permeabilized as described above. Cells were washed with PBS and mounted onto microscope slides using Citifluor (Agar Scientific). Labelled cells were viewed on a Zeiss 510-META laser scanning confocal microscope under an oil immersion × 63 objective lens.
